# Solid Fermentation Products of *Trichoderma viride* Improve Soil Aggregation, Soil Nutrient Status and Plant Growth

**DOI:** 10.3390/jof12090701

**Published:** 2026-09-19

**Authors:** Xiaowei Wei, Yuqi Pan, Mingyue Sun, Guangyuan Yao, Shihan Feng, Yidi Gai, Yize Zhao, Xuechen Yang

**Affiliations:** 1 Jilin Provincial Key Laboratory for Plant Resources Science and Green Production, Jilin Normal University, Siping 136000, China; weixiaowei@jlnu.edu.cn (X.W.); pyq20071025@163.com (Y.P.); 18543444519@163.com (M.S.); he74208645@163.com (G.Y.); 15604340110@163.com (S.F.); 15567241337@163.com (Y.Z.); 2 College of Life Sciences, Jilin University, Changchun 130000, China; gaiyd1324@mails.jlu.edu.cn; 3 State Key Laboratory of Ecological Safety and Sustainable Development in Arid Lands, Xinjiang Institute of Ecology and Geography, Chinese Academy of Sciences, Urumqi 830011, China

**Keywords:** *Trichoderma viride*, xylose residue, soil aggregates, soil nutrients, bio-organic amendment

## Abstract

Soil degradation caused by intensive agricultural management has become a major constraint on sustainable crop production, highlighting the need for multifunctional soil amendments capable of simultaneously improving soil structural quality and crop performance. In this greenhouse pot experiment, the effects of *Trichoderma viride*-fermented xylose residue on soil physical and chemical properties, aggregate stability, soil nutrient status, root development, photosynthetic characteristics, and cucumber (*Cucumis sativus* L.) growth were evaluated. The fermented amendment was incorporated into soil at application rates of 0 (CK), 0.5 (T1), 0.75 (T2), 1.0 (T3), 1.25 (T4), and 1.5% (T5) (*w*/*w*). Amendment application significantly affected soil structural characteristics and plant growth responses. Compared with the control, T3 and T4 increased the proportion of macroaggregates by 3.12% and 6.09%, respectively, whereas mean weight diameter increased by 26.81–29.76% under T3–T5. The T2 treatment produced the highest soil porosity, aeration porosity, capillary porosity, chlorophyll content, and net photosynthetic rate. Soil total nitrogen, total phosphorus, total carbon, and total organic carbon concentrations also differed significantly among treatments, while plant nitrogen and phosphorus contents were markedly enhanced under T2 and T3. Aboveground biomass increased by up to 59.80% relative to the control. Mantel analysis revealed significant associations among soil physical and chemical properties, aggregate characteristics, root morphology, plant nutrient contents, and biomass accumulation. Collectively, these findings demonstrate that *T. viride*-fermented corncob xylose residue can improve soil structural properties and promote cucumber growth under greenhouse pot conditions. Among the application rates evaluated, 0.75% provided the most balanced overall response across soil physical properties and plant performance. The present study provides a basis for the utilization of fermented lignocellulosic residues as integrated bio-organic amendments for sustainable soil management.

## 1. Introduction

Intensive agricultural management practices have accelerated soil degradation worldwide, posing a major challenge to the sustainability of crop production [1]. Continuous cultivation, excessive application of mineral fertilizers, and insufficient organic matter inputs have resulted in the deterioration of soil physical properties, including reduced aggregate stability, disrupted pore structure, and decreased nutrient retention capacity [2]. Soil aggregates are among the most important indicators of soil quality because they regulate multiple ecological functions; stable soil aggregates provide favorable microsites for organic carbon accumulation and microbial activity, thereby maintaining soil fertility and supporting plant growth [3,4]. Therefore, improving soil aggregation has become a key strategy for restoring degraded soils and promoting sustainable agricultural production.

Soil aggregation plays a fundamental role in regulating nutrient storage, transformation, and availability within soil ecosystems. Macroaggregates provide physical protection for organic matter and associated nutrients, whereas microaggregates contribute to long-term carbon stabilization and mineral-associated nutrient retention [5]. The formation and transformation of different aggregate fractions determine nutrient release dynamics and directly influence nutrient accessibility in the rhizosphere [6,7]. Previous studies have demonstrated that organic amendments can effectively improve soil aggregation and fertility [3,8]. Long-term application of organic amendments increased the proportion of macroaggregates by 8–12% compared with conventional fertilization systems, accompanied by enhanced soil organic carbon and nutrient accumulation [9,10]. Carbon-based soil amendments have also been reported to increase mean weight diameter by 33.8–47.1% and promote the formation of water-stable aggregates by more than 30% [4,11]. These findings highlight the critical role of organic carbon inputs in reconstructing soil physical environments and improving soil nutrient retention.

Improved soil aggregation can further enhance plant productivity by regulating soil nutrient cycling and root–soil interactions. Enhanced aggregate stability improves pore connectivity, reduces soil mechanical resistance, and facilitates root penetration and exploration. Consequently, improved soil structure promotes nutrient acquisition efficiency and supports plant biomass accumulation [12,13]. Previous studies have shown that organic management practices increase aggregate-associated nitrogen (N) and phosphorus (P) storage, enhance soil nutrient status, and stimulate crop growth [14,15]. However, although the relationships among soil structure, soil nutrient status, and plant productivity have been extensively investigated, the mechanisms by which specific biological amendments simultaneously regulate these processes remain insufficiently understood. The increasing accumulation of industrial organic residues has created an urgent need for sustainable strategies to achieve waste valorization and resource recycling. Xylose residue, a major by-product generated during xylose production from lignocellulosic biomass, contains abundant organic carbon, cellulose-derived compounds, hemicellulose residues, and mineral nutrients [16]. Due to its high carbon content and complex organic composition, xylose residue has potential value as a soil amendment for improving soil physical properties, increasing organic matter inputs, and promoting nutrient cycling [17]. However, direct application of untreated xylose residue to agricultural soils may result in slow decomposition, limited nutrient release, and insufficient biological activity, thereby restricting its practical utilization [18]. Therefore, developing effective biological transformation approaches to enhance the functional properties of xylose residue is essential for converting this industrial by-product into valuable agricultural resources.

Microbial solid-state fermentation provides an effective approach for improving the agricultural value of organic residues. Among beneficial microorganisms, fungi belonging to the genus *Trichoderma* have received considerable attention due to their multifunctional roles in agricultural ecosystems, including organic matter decomposition, nutrient mobilization, pathogen suppression, and plant growth promotion [13,19]. Previous studies have demonstrated that *Trichoderma* species can stimulate plant growth through the production of phytohormone-like compounds, siderophores, and extracellular metabolites, thereby enhancing root development and nutrient acquisition [20,21]. In addition, *Trichoderma* can improve soil nutrient status through biochemical processes. Organic acids produced by *Trichoderma*, such as gluconic acid, citric acid, and fumaric acid, promote mineral dissolution and nutrient mobilization. For example, *Trichoderma* application has been reported to increase soil nitrogen availability by approximately 50% and enhance soluble phosphorus content by 12%, contributing to improved soil fertility [21,22]. Beyond the effects of living microbial inoculation, increasing evidence suggests that *Trichoderma*-derived organic materials may directly contribute to soil structural improvement and nutrient regulation. Applications of *Trichoderma*-based organic fertilizers have been shown to increase the proportion of aggregates larger than 0.25 mm and enhance soil organic carbon content [19]. Similarly, substitution of chemical fertilizers with *Trichoderma asperellum* biofertilizers increased the proportion of aggregates larger than 2 mm and improved aggregate mean diameter [7]. Solid-state fermented fertilizer derived from beneficial fungi represents a novel type of bio-organic soil amendment that differs fundamentally from conventional microbial inoculants [21]. During solid-state fermentation, fungal biomass, extracellular enzymes, secondary metabolites, and transformed organic substrates accumulate within the fermentation matrix, forming a complex material with both biological and physical, as well as chemical functions [23].

Despite increasing interest in *Trichoderma* biofertilizers and soil amendments, most previous studies have focused on the effects of living *Trichoderma* inoculation, whereas the contribution of fungal solid fermentation products to soil structure reconstruction and plant growth regulation remains poorly understood [24]. In particular, it remains unclear whether xylose residue *T*. *viride* solid fermentation products can effectively enhance soil aggregate stability, improve nutrient status, and subsequently promote plant biomass production. Understanding these processes is essential for revealing the functional mechanisms of fungal-derived organic amendments and developing sustainable strategies for soil management. Therefore, this study investigated the effects of *Trichoderma viride* solid fermentation products produced from xylose residue on soil aggregation, soil physical and chemical properties, soil nutrient status, and cucumber (*Cucumis sativus* L.) growth. We hypothesized that: (1) application of *T. viride* SFPs would enhance soil aggregate formation and stability; (2) improved soil aggregation would promote nutrient retention and availability; and (3) enhanced soil structural and nutritional conditions would stimulate plant biomass accumulation. This study provides new insights into the potential of xylose residue fungal solid fermentation products as multifunctional bio-organic amendments for sustainable agricultural management.

## 2. Materials and Methods

### 2.1. Preparation of Trichoderma viride-Fermented Xylose Residue

The *Trichoderma viride* strain used in this study was originally isolated from ginseng rhizosphere soil in Jilin Province, China, and has been deposited in the China General Microbiological Culture Collection Center (CGMCC; accession No. 40034). The strain was activated on potato dextrose agar (PDA) at 28 °C for 7 d. Conidia were harvested using sterile distilled water containing 0.01% (*v*/*v*) Tween-80, and the spore concentration was determined using a hemocytometer under an inverted fluorescence microscope (Nikon Ti-s, Tokyo, Japan), and adjusted to 1.5 × 10^7^ CFU·mL^−1^ (stock conidial suspension concentration) in the culture medium. When ready for use, rinse the *T. viride* conidia with sterile water, collect the conidial suspension, and adjust the conidial concentration to 1.5 × 10^8^ CFU L^−1^ (final inoculum concentration).

The xylose residue used in this study is an industrial by-product from a lignocellulosic biorefinery, produced using corn cob as the raw material for commercial xylose manufacturing. After dilute acid hydrolysis of corn cob, xylose is extracted via evaporation, crystallization and purification, and the remaining solid waste after xylose separation is the test xylose residue, which was directly obtained from Siping Lyujian Biotechnology Co., Ltd. (Siping, China). Its basic properties are: total organic carbon 47.23 g·kg^−1^, total nitrogen 6.8 g·kg^−1^, and total phosphorus 1.2 g·kg^−1^, pH 4.72. Before fermentation, the residue was sterilized in a vertical autoclave at 121 °C for 20 min and cooled under aseptic conditions.

Solid-state fermentation was conducted using sterilized corncob xylose residue as the principal substrate. For each 40 g of substrate (dry weight), 0.28 g glucose and 0.164 g urea were added as supplementary carbon and nitrogen sources, respectively. A total of 26.19 mL of Mandels nutrient solution (pH 8.7) (2.0 g L^−1^ KH_2_PO_4_, 0.3 g L^−1^ MgSO_4_, 0.3 g L^−1^ CaCl_2_, 5.0 mg L^−1^ FeSO_4_, 1.4 mg L^−1^ ZnSO_4_, and 1.6 mg L^−1^ MnSO_4_. All reagents and chemicals were purchased from Sinopharm Chemical Reagent Co., Ltd., Shanghai, China, and 5 mL of *T. viride* inoculum (1.5 × 10^8^ CFU L^−1^) was added to each 40 g batch of xylose residue (dry weight). The substrate was thoroughly mixed to ensure uniform distribution of moisture, nutrients, and inoculum. Fermentation was carried out in sterile culture bottles at 28 °C for 15 d under aseptic conditions. The substrate layer was maintained at approximately 5 cm, and the material was manually mixed every 24 h to improve aeration and maintain homogeneous fermentation. After fermentation, the fermented product was directly employed as the experimental soil amendment without additional drying treatment.

### 2.2. Experimental Soil and Greenhouse Pot Experiment

The pot experiment was conducted at the Jilin Provincial Key Laboratory for Plant Resources Science and Green Production (42°51′ N, 124°31′ E), China. The experimental soil was black soil collected from the experimental field. Prior to use, the soil was sterilized in a vertical autoclave at 121 °C for 20 min. The basic physical and chemical properties of the soil were: pH of 6.89, EC of 64.12 μS·cm^−1^, total N of 2.42 g·kg^−1^, total P of 112.4 mg·kg^−1^, and organic C of 8.37 g·kg^−1^. The study employed a completely randomized design with six amendment treatments. The pots had dimensions of 18 cm in diameter and 22 cm in height; the substrate was then mixed with varying amounts of the solid fermentation product of *Trichoderma viride*. The mixture was then added to the pots, with each pot containing 3.0 kg of soil; the *Trichoderma viride* solid-state fermentation substrate was added at rates of 0.0% (CK), 0.5% (T1), 0.75% (T2), 1.0% (T3), 1.25% (T4), and 1.5% (T5) (*w*/*w*), and each treatment was replicated six times.

These cucumber seeds (Jinyan No. 4; Cangzhou Jinkelifeng Seedling Co., Ltd., Cangzhou, China) were first germinated in a seedling tray, and after germination, they were transplanted into experimental treatment pots. Watering was performed once every three days, with 100 mL of water applied to each pot at a time. The experiment was conducted in a greenhouse, with climate conditions set to a daytime/nighttime temperature of 27 °C/23 °C, a light/dark cycle of 16 h/8 h, light intensity of 1200 μmol·m^−2^·s^−1^, and relative humidity of 45%. Adequate soil moisture was maintained throughout the cucumber seedling cultivation period. The cucumber seedlings were grown to the five-leaf stage, at which point soil physical and chemical properties and plant traits were measured.

### 2.3. Soil Sampling and Physical and Chemical Analyses

After harvest, soil from each pot was gently passed through a 2-mm sieve to remove roots and coarse debris while minimizing disturbance of the natural aggregate structure. The processed soil was then divided into two subsamples.

The first subsample was air-dried, finely ground, and passed through a 100-μm sieve for determination of soil chemical properties. Another subsample was reserved exclusively for aggregate-size analysis and was not passed through the 100-μm sieve before wet sieving. Soil pH and EC were measured in a 1:5 (*w*/*v*) soil–water suspension after shaking for 30 min and standing for an additional 30 min. Soil pH was determined using a PB10 pH meter (Sartorius, Göttingen, Germany), whereas EC was measured using a DDS-307 conductivity meter (Leici, Shanghai, China). Soil bulk density and soil moisture were determined using the ring-knife method with 100-cm^3^ stainless-steel cylinders. Samples were dried at 105 °C to constant mass before calculation; total nitrogen and total carbon were determined using an elemental analyzer (vario EL cube, Elementar, Langenselbold, Germany). Total phosphorus was measured using a SmartChem 450 discrete chemical analyzer (AMS Alliance, Pavia, Italy). Total organic carbon was determined using a Vario TOC analyzer (Elementar Analysensysteme GmbH, Langenselbold, Germany); carbonates (inorganic carbon) were removed from the soil using the dilute hydrochloric acid (HCl) acid-washing method.

Soil bulk density was determined using the core method. Soil samples were collected with a stainless-steel core ring of known volume (100 cm^3^) and oven-dried to a constant weight. The bulk density was calculated as

(1) $$d_{v}=\frac{W-W_{ring}}{V}$$

where *d_v_* is the soil bulk density (g cm^−3^); *W* is the combined weight of the oven-dried soil and the core ring (g); *W_ring_* is the weight of the empty core ring (g); and *V* is the volume of the core ring (100 cm^3^).

Soil porosity was determined using the core method. A piece of filter paper was placed on one end of the core ring, which was then positioned in a shallow tray containing a thin layer of water. The core was allowed to absorb water by capillary action. When the filter paper first became moist, the core was removed, gently wiped dry, and immediately weighed (*W*_1_). The core was subsequently immersed again until the filter paper on the upper surface became completely saturated, after which it was weighed (*W*_2_). Finally, the core containing the soil sample was oven-dried to a constant weight and weighed (*W*_3_).

Capillary porosity and total porosity were calculated as follows:

(2) $$Capillary\;porosity\;(\%)=\frac{W_{1}-W_{3}}{V}\times 100$$

(3) $$Soil\;porosity\;(\%)=\frac{W_{2}-W_{3}}{V}\times 100$$

where *V* is the volume of the core ring (100 cm^3^).

Soil aeration porosity (%) was calculated as

Soil aeration (%) = Soil porosity (%) − Volumetric water content (%)(4)

### 2.4. Soil Aggregate Analysis

Soil aggregates were fractionated by wet sieving. Briefly, approximately 200 g of fresh soil was pre-wetted for 5 min and separated into macroaggregates (>250 μm), microaggregates (53–250 μm), and silt-clay (<53 μm) using 250- and 53-μm sieves. Aggregate-size distribution was determined separately using 50 g of air-dried soil following the same wet-sieving procedure. The collected fractions were dried at 65 °C and weighed to calculate their mass distribution. Soil aggregate stability was evaluated using the mean weight diameter (MWD) and geometric mean diameter (GMD), which were calculated as follows:

(5) $$MWD=\sum_{i=1}^{n}\bar{D}W_{i}$$

(6) $$GMD=exp\left[\sum_{i=1}^{n}\left(W_{i}\times \ln\bar{D}\right)\right]$$

where $\bar{D}$ is the mean diameter (mm) of each aggregate-size fraction, and $W_{i}$ is the proportion (%) of each soil aggregate fraction.

### 2.5. Plant Measurements

Net photosynthetic rate (P_n_, μ mol m^−2^ s^−1^) was measured using a CIRAS-3 portable photosynthesis system (PP Systems, Amesbury, MA, USA) at 25 °C, with a CO_2_ concentration of 400 μ mol mol^−1^ and a 500 μ mol s^−1^ flow rate. The photosynthetic photon flux density (PPFD) was set to 1000 μ mol m^−2^ s^−1^ [25]. For chlorophyll determination, three fully expanded leaves were collected from each pot and combined to form one composite sample. Consequently, six independent composite samples (*n* = 6) were analyzed for each treatment. Chlorophyll content (mg g^−1^ fresh weight) was analyzed by extracting pigments from 0.1 g of fresh leaf samples with ethanol. The absorbance of the resulting solution was measured at 645 and 663 nm using a UV–visible spectrophotometer (UVmini-1240, Shimadzu, Kyoto, Japan). Chlorophyll concentration was then determined according to the equations reported by Wei et al. (2024) [26].

(7) $${Chlorophyll}_{a}=12.56\times A_{665}-2.71\times A_{645}$$

(8) $${Chlorophyll}_{b}=22.65\times A_{645}-4.35\times A_{663}$$

(9) $${Chlorophyll}_{a+b}={Chlorophyll}_{a}+{Chlorophyll}_{b}$$

Root systems of cucumber plants were washed, scanned with an Epson 11000 scanner (Epson America, Inc., Los Alamitos, CA, USA), and analyzed using WinRHIZO 2016b (Regent Instrument, Quebec, QC, Canada) to determine root length, surface area, volume, and tip number. Within each pot, three cucumber plants were measured and averaged to generate one independent pot-level observation. Aboveground biomass (AGB) and belowground biomass (BGB) were determined after drying samples at 65 °C to constant weight. Plant total nitrogen and total phosphorus contents were measured using the same analytical procedures described for soil samples.

### 2.6. Statistical Analysis

Each pot was regarded as one independent experimental unit. Data were analyzed using one-way analysis of variance (ANOVA) with amendment application rate as the fixed factor. Normality and homogeneity of variance were evaluated using the Kolmogorov–Smirnov and Levene tests, respectively. When treatment effects were significant (*p* < 0.05), means were separated using Tukey’s honestly significant difference (HSD) test. Pearson correlation analysis was first performed to evaluate pairwise relationships among soil physicochemical properties (pH, EC, soil bulk density, soil moisture, TN, TP, TC, and TOC), soil aggregate characteristics (soil macroaggregates, soil microaggregates, and silt-clay aggregates), soil pore characteristics (soil porosity, soil aeration, and capillary porosity), root morphological traits (total root length, total root surface area, total root projected area, average root diameter, and root volume), photosynthetic-related traits (chlorophyll a, chlorophyll b, chlorophyll a + b, and net photosynthetic rate), and plant nutrient contents (plant nitrogen and plant phosphorus). The resulting pairwise Pearson’s r values were used to construct the correlation matrix. Mantel tests were subsequently performed to examine the relationships of aboveground biomass (AGB) and belowground biomass (BGB) with the above soil and plant variables. For each variable, a distance matrix was constructed based on measurements across the experimental samples, and its relationship with the corresponding AGB or BGB distance matrix was evaluated using the Mantel test. Mantel’s r was used to represent the strength of the association, and statistical significance was assessed by permutation tests. All statistical analyses were performed using R software (v4.5.1), and graphical visualization was generated using the ggplot2 (2.2.1) package.

## 3. Results

### 3.1. Soil Physical and Chemical Properties

**Figure 1:**
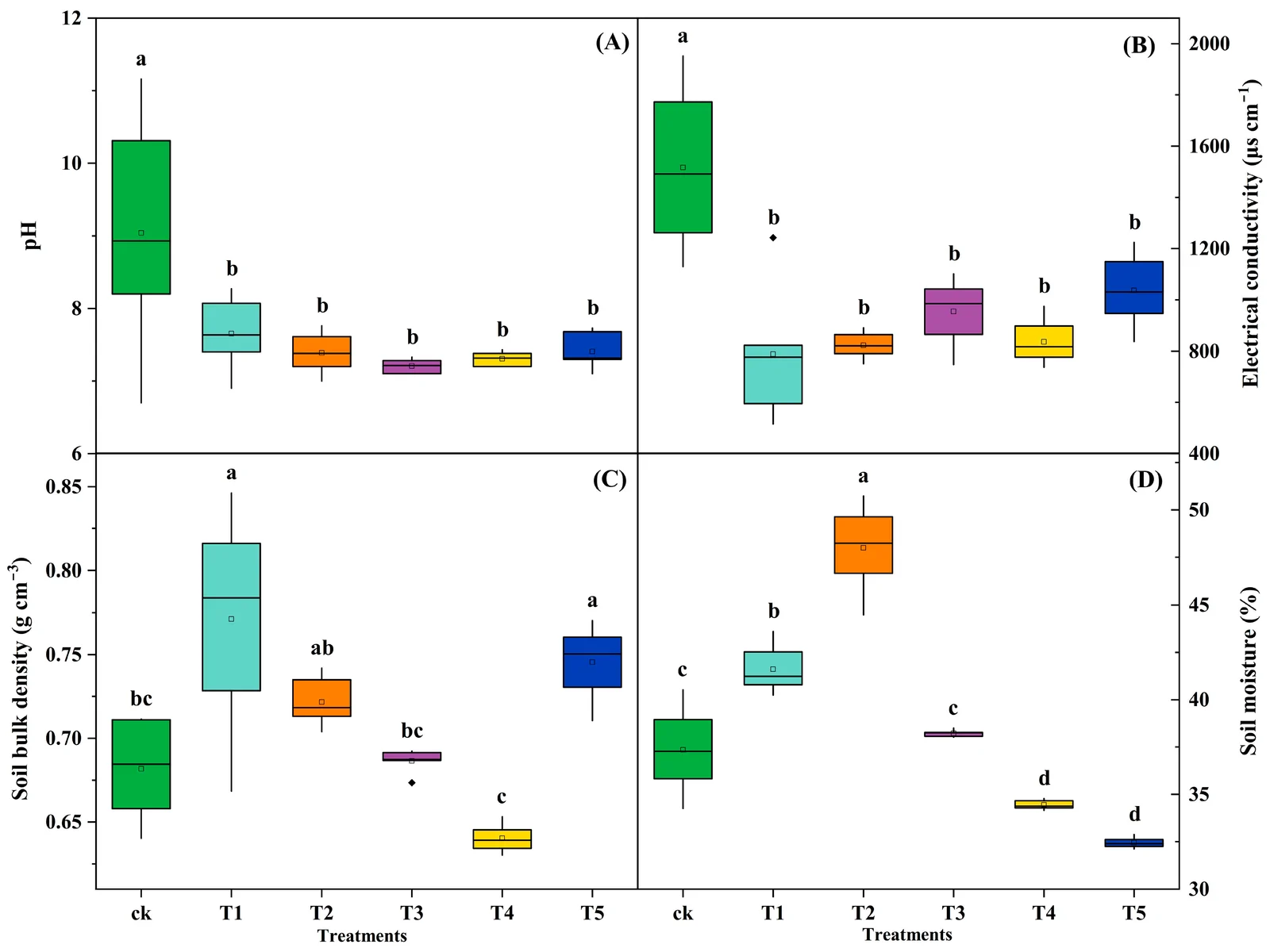
Effects of *T. viride* solid-state fermentation substrate application on soil pH (**A**), electrical conductivity (**B**), soil bulk density (**C**), and soil moisture (**D**). *T. viride* solid-state fermentation substrate was added at rates of 0.0% (CK), 0.5% (T1), 0.75% (T2), 1.0% (T3), 1.25% (T4), and 1.5% (T5). Statistically significant differences among treatments are indicated by different lowercase letters based on the Tukey test (*p* < 0.05; *n* = 6).

**Figure 2:**
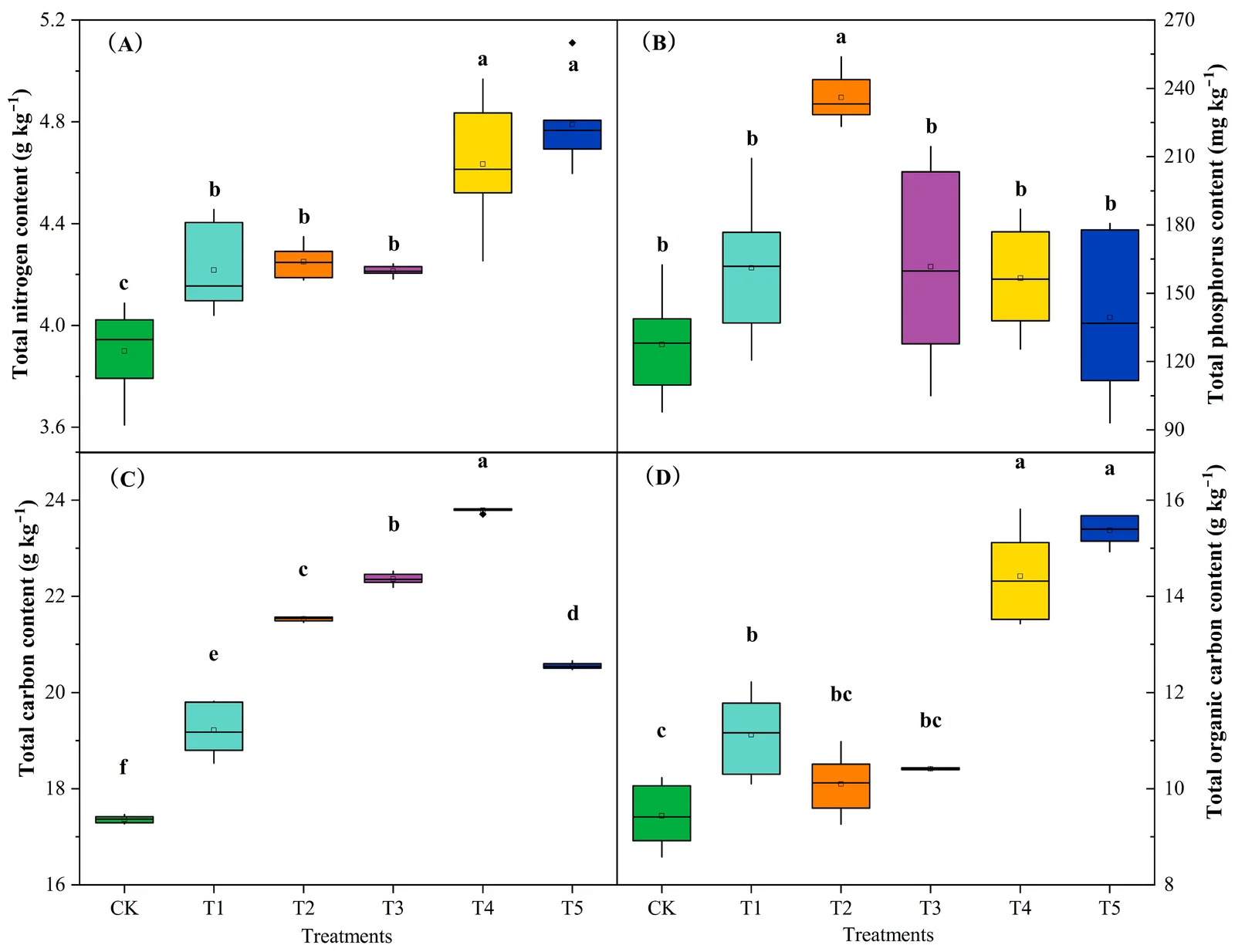
Effects of *T. viride* solid-state fermentation substrate application on total soil nitrogen content (**A**), total phosphorus content (**B**), total carbon content (**C**), and total organic carbon content (**D**). *T. viride* solid-state fermentation substrate was added at rates of 0.0% (CK), 0.5% (T1), 0.75% (T2), 1.0% (T3), 1.25% (T4), and 1.5% (T5). Statistically significant differences among treatments are indicated by different lowercase letters based on the Tukey test (*p* < 0.05; *n* = 6).

Application of *T. viride*-fermented xylose residue significantly altered several soil physical and chemical properties (Figure 1 and Figure 2). Soil pH differed significantly among treatments (Figure 1A). Compared with the control (T0), all amendment treatments resulted in lower final soil pH values, although the magnitude of the response varied with application rate. Electrical conductivity also responded significantly to amendment application (Figure 1B). The T2 and T5 treatments exhibited significantly lower EC values than the control, whereas intermediate application rates (T3 and T4) maintained comparatively higher EC values. Bulk density showed a nonlinear response to amendment addition (Figure 1C). The highest values were observed under T1 and T5, whereas T2–T4 maintained relatively lower bulk density. In contrast, soil moisture differed markedly among treatments (Figure 1D), with T2 exhibiting the highest moisture content and T5 the lowest.

The amendment also significantly influenced soil nutrient status (Figure 2). Total soil N content increased progressively with increasing amendment rate and reached its highest value under T5, representing a 22.82% increase relative to the control (Figure 2A). Total soil phosphorus responded differently, with the greatest concentration observed under T2, which exceeded the control by 85.01% (Figure 2B). STC and SOC also varied significantly among treatments. TC reached its maximum under T4 (Figure 2C), whereas SOC was significantly higher under T4 and T5 than under the remaining treatments (Figure 2D).

### 3.2. Soil Aggregate Size Distribution and Structural Stability

**Figure 3:**
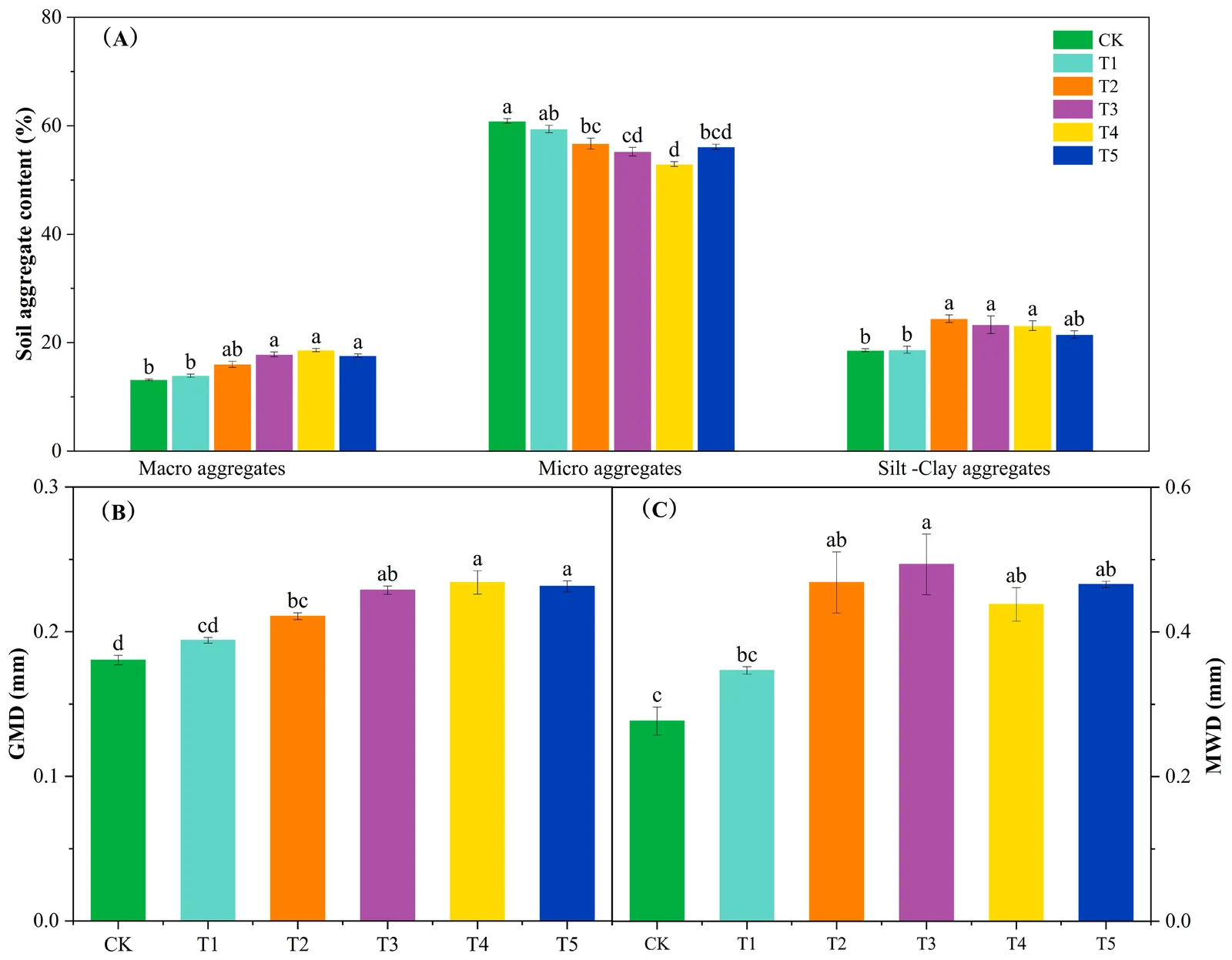
Effects of *T. viride* solid-state fermentation substrate application on the proportion of soil aggregates (**A**), the mean weight diameter (GMD) (**B**), and geometric mean diameter (MWD) (**C**) of soil aggregates. *T. viride* solid-state fermentation substrate was added at rates of 0.0% (CK), 0.5% (T1), 0.75% (T2), 1.0% (T3), 1.25% (T4), and 1.5% (T5). Statistically significant differences between treatments are indicated by different lowercase letters based on the Tukey test (*p* < 0.05; *n* = 6).

Application of *T. viride*-fermented xylose residue significantly modified aggregate-size distribution and aggregate stability (Figure 3). The proportion of macroaggregates (>250 μm) increased following amendment application, with the greatest increases observed under T3 and T4, representing improvements of 3.12% and 6.09%, respectively, compared with the control (Figure 3A). Conversely, microaggregate proportions were highest in CK and T1 and declined at intermediate amendment rates. The relative abundance of the silt–clay fraction was significantly greater in T2–T4 than in the remaining treatments.

**Figure 4:**
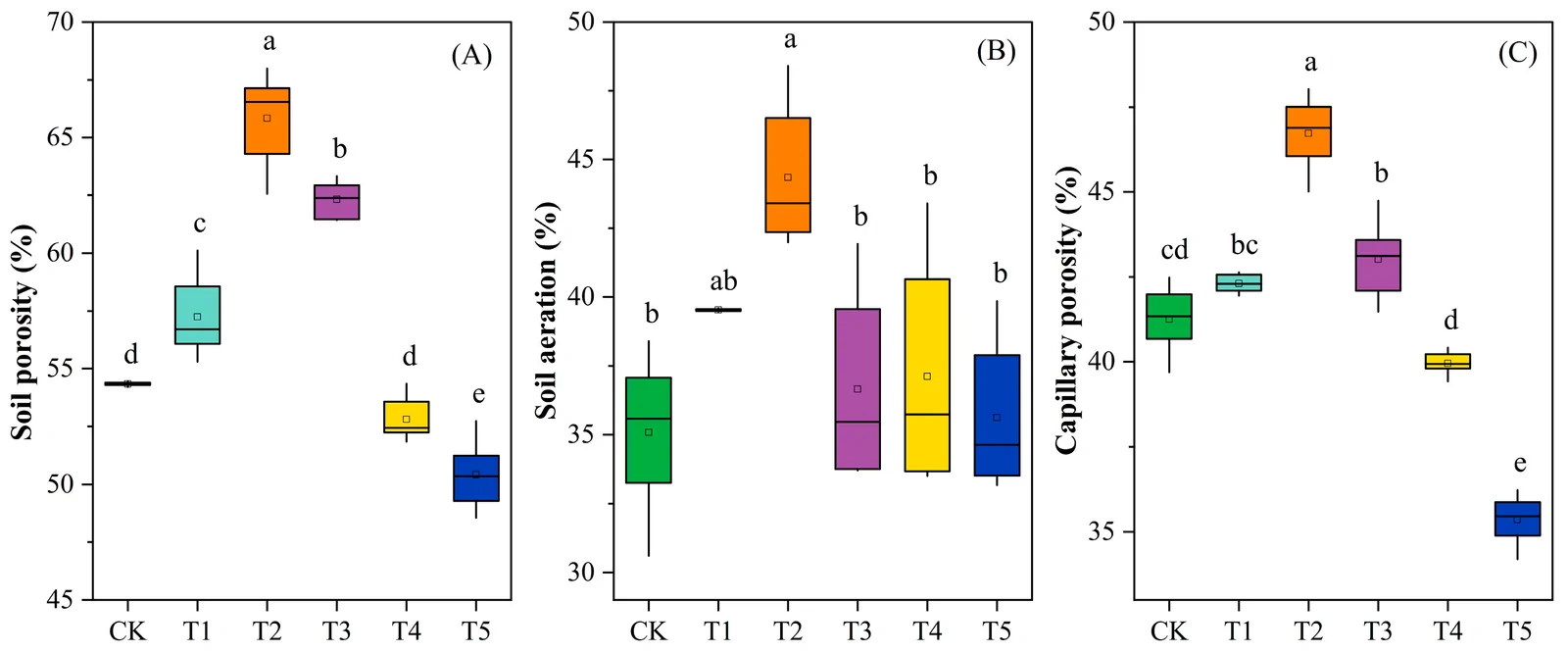
Effects of *T. viride* solid-state fermentation substrate application on soil porosity (**A**), soil aeration (**B**), and capillary porosity (**C**). *T. viride* solid-state fermentation substrate was added at rates of 0.0% (CK), 0.5% (T1), 0.75% (T2), 1.0% (T3), 1.25% (T4), and 1.5% (T5). Statistically significant differences between treatments are indicated by different lowercase letters based on the Tukey test (*p* < 0.05; *n* = 6).

Aggregate stability indices also responded positively to amendment application. Geometric mean diameter (GMD) increased significantly under T3 relative to CK and T1 (Figure 3B), whereas no significant differences were detected among T2, T4, and T5. Mean weight diameter (MWD) increased significantly under T3–T5, with improvements ranging from 26.81% to 29.76% relative to the control (Figure 3C). The amendment also affected soil pore characteristics (Figure 4). Soil porosity, aeration porosity, and capillary porosity all reached their highest values under T2. Compared with the control, soil porosity increased by 21.17%, aeration porosity by 26.41%, and capillary porosity by 32.17%. Higher amendment rates did not further improve these parameters.

### 3.3. Root Morphology

Root morphological traits differed significantly among amendment treatments (Table 1). Total root length was greatest under T2, reaching 412.95 cm, which was significantly higher than that of the control and most other treatments. Root surface area exhibited a similar response, with the maximum value observed under T3, followed by T2. Total projected root area and root volume were significantly greater under both T2 and T3 than under the remaining treatments, indicating enhanced root system development at moderate amendment rates. Average root diameter also varied significantly among treatments, reaching its highest value under T3. However, increasing amendment rates beyond 1.0% did not produce additional improvements in root morphological characteristics.

### 3.4. Plant Photosynthetic Characteristics and Nutrient Contents

**Figure 5:**
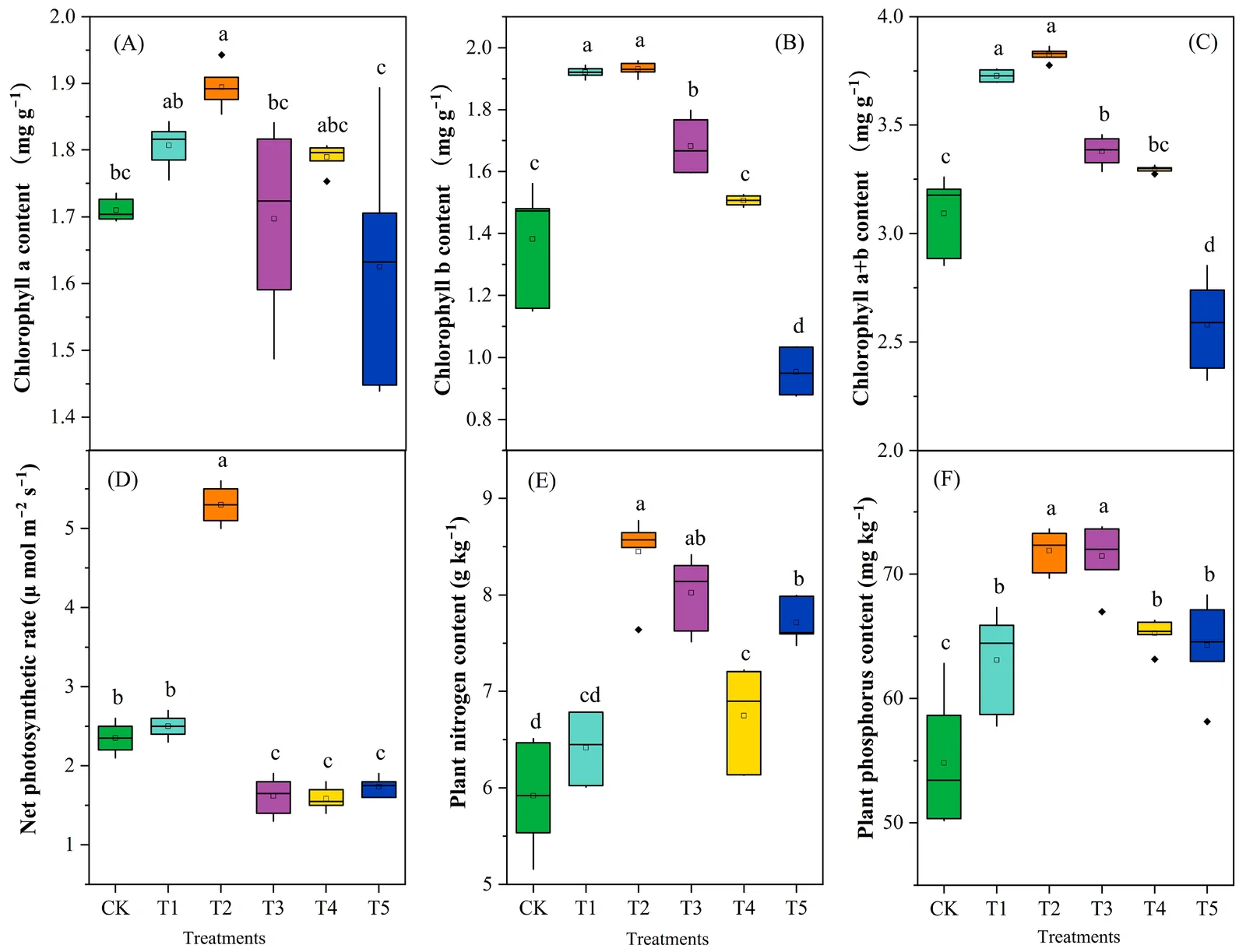
Effects of *T. viride* solid-state fermentation substrate application on Chlorophyll a content (**A**), Chlorophyll b content (**B**), Chlorophyll a + b content (**C**), Net photosynthetic rate (**D**), Plant nitrogen content (**E**) and Plant phosphorus content (**F**). *T. viride* solid-state fermentation substrate was added at rates of 0.0% (CK), 0.5% (T1), 0.75% (T2), 1.0% (T3), 1.25% (T4), and 1.5% (T5). Statistically significant differences between treatments are indicated by different lowercase letters based on the Tukey test (*p* < 0.05; *n* = 6).

Amendment application affected photosynthetic characteristics and plant nutrient contents (Figure 5). The accumulation of photosynthetic pigments showed that the T2 treatment significantly promoted the accumulation of chlorophyll a and chlorophyll b. The contents of both pigments were significantly higher under the T2 treatment than under the CK and T5 treatments. Specifically, chlorophyll a content in the T2 treatment increased by 10.80% and 16.57% compared with the CK and T5 treatments, respectively (Figure 5A). Chlorophyll b content in the T2 treatment increased by 39.72% compared with the CK treatment (Figure 5B). The total chlorophyll a + b content was also significantly higher in the T2 treatment than in all other treatments (Figure 5C). The net photosynthetic rate was significantly higher in the T2 treatment than in all other treatments, whereas no significant differences were observed among the T3, T4, and T5 treatments (Figure 5D). Plant nutrient accumulation also responded positively to amendment application. Plant total nitrogen and total phosphorus contents were significantly higher under T2 and T3 than under the remaining treatments. Relative to the control, plant nitrogen increased by 42.71% under T2 and 35.52% under T3, whereas plant phosphorus increased by 31.15% and 30.36%, respectively (Figure 5E,F).

### 3.5. Plant Biomass Accumulation

**Figure 6:**
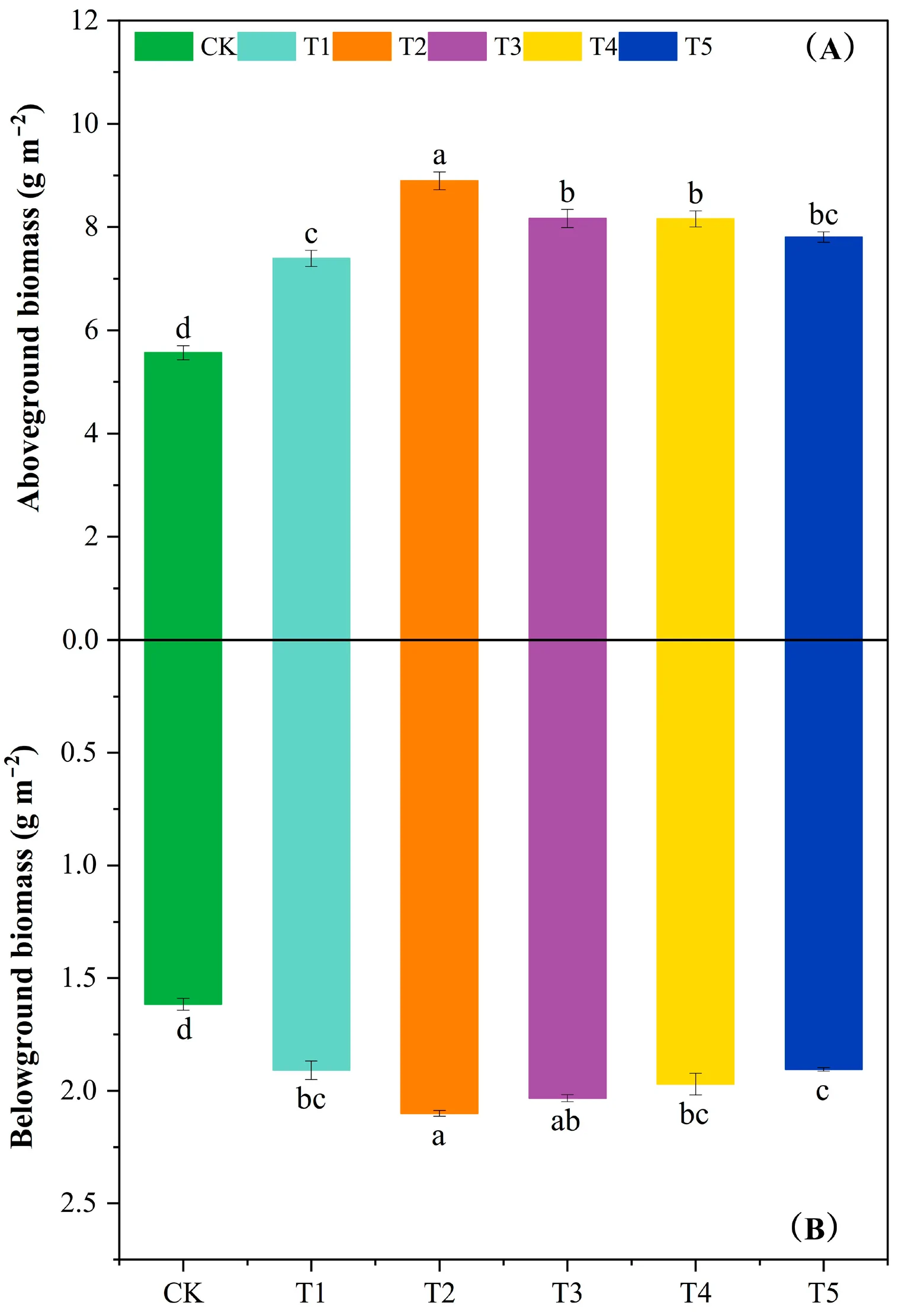
Effects of *T. viride* solid-state fermentation substrate application on aboveground biomass (**A**) and belowground biomass (**B**). *T. viride* solid-state fermentation substrate was added at rates of 0.0% (CK), 0.5% (T1), 0.75% (T2), 1.0% (T3), 1.25% (T4), and 1.5% (T5). Statistically significant differences between treatments are indicated by different lowercase letters based on the Tukey test (*p* < 0.05; *n* = 6).

Aboveground and belowground biomass responded significantly to the *T. viride* solid-state fermentation substrate treatments. Compared with the CK treatment, aboveground biomass was substantially enhanced by the *T. viride* solid-state fermentation substrate addition, with increases of 59.80%, 46.69%, and 46.58% under T2, T3, and T4 treatments, respectively (Figure 6A). Belowground biomass followed a similar pattern. Root biomass was significantly greater under T2 and T3 than under the control, whereas higher amendment rates did not further enhance biomass accumulation. (Figure 6B).

### 3.6. Relationships Among Soil Properties, Root Traits, Nutrient Status, and Biomass

**Figure 7:**
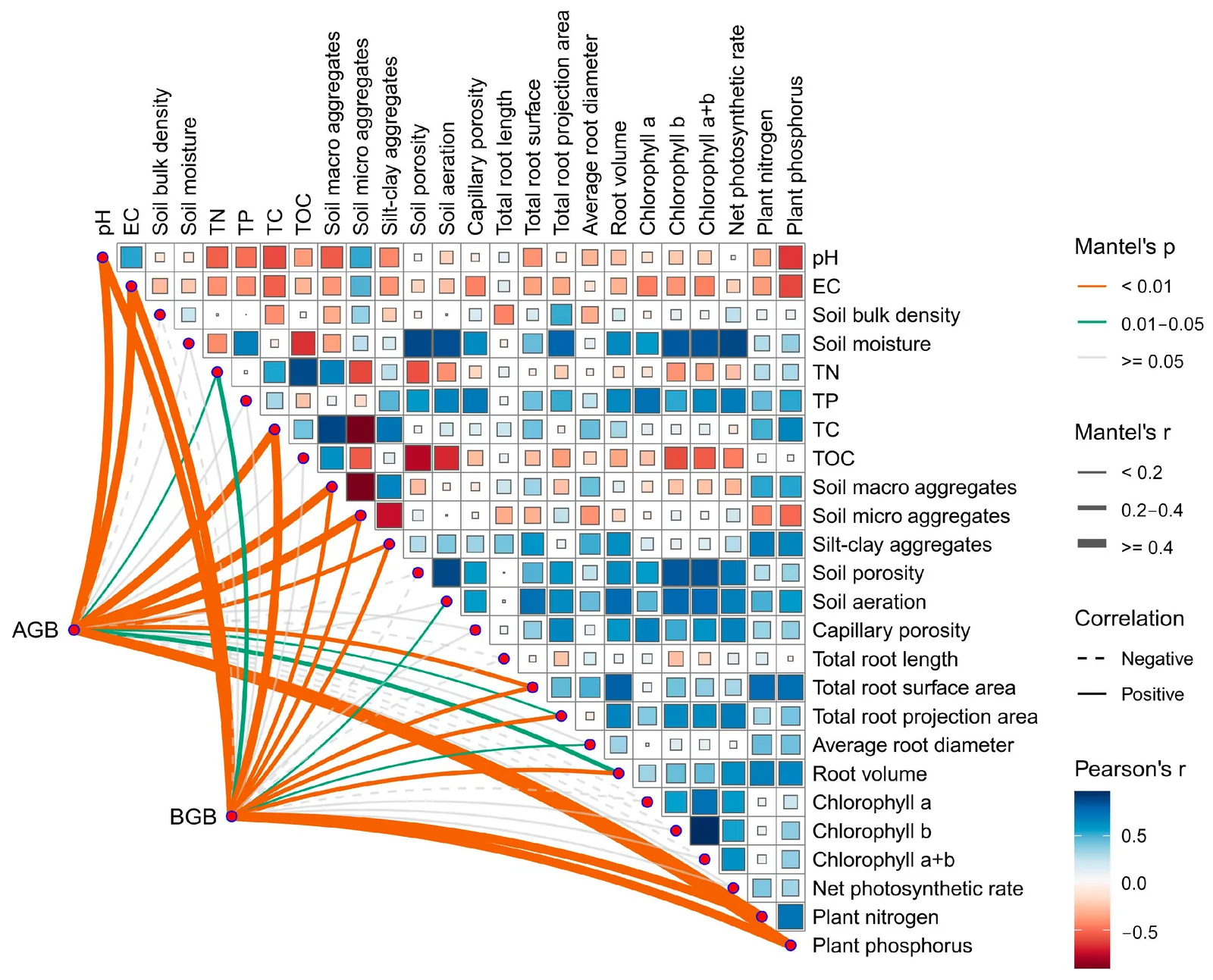
Relationships among aboveground biomass (AGB), belowground biomass (BGB), soil properties, root traits, photosynthetic characteristics, and plant nutrient status based on Pearson correlation analysis and Mantel tests. The triangular matrix shows pairwise Pearson correlations among soil physicochemical properties, soil aggregate characteristics, soil pore characteristics, root morphological traits, photosynthetic-related parameters, and plant nutrient contents. Blue and red squares indicate positive and negative Pearson correlations, respectively, with color intensity representing the magnitude of Pearson’s r. Mantel tests were performed separately to evaluate the relationships of AGB and BGB with individual soil and plant variables. The width of the connecting lines represents the magnitude of Mantel’s r, while line color indicates the significance level (*p* < 0.01, 0.01 ≤ *p* < 0.05, and *p* ≥ 0.05). Solid and dashed lines indicate positive and negative relationships, respectively.

Pearson correlation and Mantel analyses revealed coordinated relationships among soil properties, root traits, plant nutrient status, and biomass accumulation (Figure 7). The Pearson correlation matrix showed multiple associations among soil physicochemical properties, soil aggregate characteristics, pore characteristics, root morphological traits, photosynthetic parameters, and plant nutrient contents. In particular, several soil nutrient and structural variables were closely correlated with root development and plant physiological traits.

Mantel tests further demonstrated that AGB and BGB were significantly associated with multiple soil and plant variables. Significant associations were observed between biomass and soil properties, including pH, electrical conductivity, soil nutrient status, and aggregate characteristics. Biomass was also closely associated with root morphological traits and plant N and P contents. These results indicate that biomass accumulation under the experimental conditions was jointly associated with soil physicochemical conditions, soil structural properties, root development, and plant nutrient status.

## 4. Discussion

### 4.1. Effects of T. viride-Fermented Xylose Residue on Soil Structure and Nutrient Status

Soil aggregation is widely recognized as a key indicator of soil structural quality because it influences pore architecture, water retention, aeration, organic matter stabilization, and nutrient storage. According to the aggregate hierarchy theory proposed by Adnan et al. [27] and further developed by Hamza et al. [28], macroaggregates are formed through the binding of microaggregates by fungal hyphae, plant roots, and microbial-derived organic materials, and they play a pivotal role in stabilizing soil organic carbon (SOC) and maintaining soil structural stability. Consequently, organic amendments and beneficial microorganisms, particularly *Trichoderma* spp., have been extensively investigated as effective strategies for improving degraded soils through aggregate formation and carbon accumulation [4,29]. Previous studies consistently demonstrated that fungal inoculation or fermented organic amendments increase macroaggregate formation, enhance aggregate stability, and promote SOC sequestration by stimulating microbial activity and supplying organic binding agents [30].

In the present study, application of *T. viride*-fermented xylose residue significantly altered aggregate-size distribution and increased both GMD and MWD, indicating an overall improvement in soil structural stability. Moderate amendment rates also produced higher soil porosity, aeration porosity, and capillary porosity, demonstrating that the fermented amendment influenced multiple aspects of soil physical quality rather than aggregate stability alone. The enhanced aggregation can be attributed to the combined effects of *T. viride* and fermentation-derived organic matter. During solid-state fermentation, *T. viride* secretes cellulolytic and ligninolytic enzymes that convert lignocellulosic components of xylose residue into more labile carbon fractions, which stimulate microbial growth and extracellular polymeric substance (EPS) production [6,13]. Meanwhile, fungal hyphae physically enmesh soil particles and microaggregates, while microbial metabolites function as transient binding agents that promote macroaggregate formation [31,32]. The concomitant increases in TC and TOC further suggest that newly formed macroaggregates physically protected organic carbon from rapid mineralization, thereby reinforcing aggregate stability through positive feedback between SOC accumulation and aggregate formation [1,10]. Interestingly, the improvement in soil aggregate stability did not completely correspond with improvements in soil physical quality [30]. Although T3–T5 generally produced greater increases in macroaggregates and aggregate stability, the T2 treatment exhibited the highest porosity and aeration, suggesting that moderate amendment application optimized pore connectivity more effectively than higher application rates. This divergence suggests that different components of soil structural quality responded differently to amendment rate. Moderate amendment application appeared to provide a more favorable pore architecture than higher application rates, whereas greater amendment inputs primarily increased aggregate stability and carbon accumulation.

The initial soil pH measured before the experiment differed from the pH determined at the end of the greenhouse experiment. The initial value represented the physical and chemical characteristics of the soil before sterilization and cultivation, whereas the latter reflected the combined effects of soil sterilization, amendment incorporation, greenhouse incubation, and plant growth. Because pH was not monitored continuously during these stages, the present study cannot distinguish the relative contributions of these factors. Consequently, the mechanisms responsible for the observed pH shift remain uncertain and require further investigation.

### 4.2. Associations Between Soil Improvement and Plant Growth

Improved soil physical and chemical properties and aggregate stability were accompanied by pronounced increases in plant growth, indicating that the benefits of the *T. viride* solid-state fermentation substrate extended from soil improvement to plant physiological performance [33,34]. Compared with the control, T2 and T3 significantly enhanced root length, surface area, projected area, average diameter, and volume, together with plant nitrogen and phosphorus accumulation, chlorophyll content, net photosynthetic rate, and biomass. T2 consistently produced the strongest responses, suggesting that moderate substrate application created the most favorable rhizosphere conditions. Meanwhile, higher application rates did not result in proportional improvements in plant growth. These observations indicate that moderate amendment application provided the most favorable balance between soil physical conditions and plant responses. Biomass enhancement was not attributable solely to increased nutrient supply, but rather to coordinated improvements in root architecture, nutrient acquisition, and photosynthetic capacity [16,35]. Root architecture is a major determinant of nutrient acquisition because it governs the volume of soil explored by plants [30]. The improved root morphology under T2 and T3 was likely associated with enhanced soil aggregation and pore structure. Stable macroaggregates improve pore continuity and aeration while reducing mechanical resistance, thereby facilitating root penetration and elongation. In addition to these physical effects, *Trichoderma* can directly stimulate root development. Root colonization by *Trichoderma* spp. has been reported to promote lateral root formation and branching through auxin-related compounds, peptides, volatile organic compounds, and other bioactive metabolites [13,24,36]. The substantial increases in total root length, surface area, and root volume observed here therefore likely reflect synergistic effects of improved soil structure and *T. viride*-mediated root stimulation.

The expanded root system subsequently enhanced nutrient acquisition. Plant N and P accumulation increased markedly under T2 and T3, consistent with both greater soil nutrient status and higher root absorptive capacity. Increased root length and surface area enlarge the soil–root interface and improve nutrient interception, particularly for relatively immobile phosphorus. Meanwhile, *Trichoderma* spp. can mobilize nutrients by releasing organic acids, phosphatases, siderophores, and extracellular enzymes that promote mineral solubilization and nutrient transformation in the rhizosphere [37,38]. Accordingly, the greater plant P accumulation under T2 likely resulted from the combined effects of increased soil phosphorus availability and improved root exploration, whereas enhanced N accumulation reflected both improved soil nutrient status and greater uptake efficiency [21]. Enhanced nutrient acquisition contributed to improved photosynthetic performance. Nitrogen is essential for chlorophyll, Rubisco, and photosynthetic enzymes, whereas phosphorus supports ATP production, electron transport, and carbon metabolism [25]. Increased nitrogen and phosphorus uptake therefore provides a physiological basis for enhanced chlorophyll synthesis and photosynthetic activity [21,25,39]. In this study, chlorophyll a, chlorophyll b, total chlorophyll, and net photosynthetic rate all reached their highest values under T2. Similar effects have been reported following *Trichoderma* inoculation, including increased chlorophyll content, stomatal conductance, carbon assimilation, and water-use efficiency [40,41]. These responses are generally attributed to improved mineral nutrition, hormonal regulation, and enhanced root activity [42]. Thus, the superior photosynthetic performance under T2 likely resulted from the combined effects of improved nutrient acquisition and *T. viride*-mediated physiological stimulation.

The substantial increase in aboveground biomass under T2 likely resulted from the combined effects of improved soil physical conditions, enhanced root development, and greater plant nutrient accumulation. Mantel analysis further demonstrated significant associations among soil structural characteristics, plant nutrient contents, root morphology, and biomass production. However, Mantel analysis evaluates statistical associations rather than causal relationships. Therefore, the present study supports the existence of coordinated responses across the soil–plant system but does not establish a causal pathway linking soil aggregation to biomass production. Overall, the intermediate application rate (0.75%, T2) provided the most balanced response across soil physical properties and plant performance. Although higher amendment rates further increased some aggregate and carbon-related parameters, they did not consistently improve photosynthesis or biomass accumulation. These findings suggest that excessive amendment application may not provide additional agronomic benefits under the conditions of the present greenhouse experiment.

The significant associations among soil properties, aggregate characteristics, root traits, plant nutrient contents, and biomass suggest that the observed plant responses were closely linked to coordinated changes in the soil–plant system. However, because the present study quantified total soil nutrient pools rather than plant-available nutrient fractions and did not directly measure fungal colonization, extracellular enzyme activities, microbial metabolites, or nutrient transformation processes, these relationships should not be interpreted as proof of a specific mechanistic pathway. In addition, the absence of non-fermented xylose residue and nutrient-equivalent controls prevents the effects of fermentation itself from being separated from those of organic substrate and nutrient addition.

## 5. Conclusions

This study provides evidence that *Trichoderma viride*-fermented xylose residue can function as a multifunctional bio-organic amendment under greenhouse pot conditions, with measurable effects on soil structural properties and cucumber performance. Amendment application improved aggregate stability and altered soil physical and chemical characteristics, while the 0.75% treatment produced the most balanced overall response across soil porosity, root development, plant N and P accumulation, photosynthetic performance, and biomass production. Higher application rates further enhanced some aggregate and carbon-related indicators, but these responses did not consistently translate into additional plant growth benefits, indicating that the agronomic response was not simply proportional to amendment rate. Overall, the 0.75% application rate achieved the best balance between soil structural improvement and plant physiological performance. From an applied perspective, the results support the potential use of *T. viride*-fermented xylose residue as a value-added amendment for improving soil structural quality while promoting cucumber growth, thereby providing a possible route for the agricultural utilization of industrial lignocellulosic residues. Among the application rates tested, 0.75% appears to be the most promising rate for further evaluation. Future research should incorporate appropriate process controls, direct measurements of plant-available nutrients and microbial functional traits, and long-term field experiments to determine the persistence, mechanism, and practical agronomic value of this amendment.

## Figures and Tables

**Table 1:** Comparison of the total root length, total root surface, total root projection area, average root diameter and root volume in *Cucumber plants* under different *T. viride* solid-state fermentation substrate application treatments (means ± SE, *n* = 6). *T. viride* solid-state fermentation substrate was added at rates of 0.0% (CK), 0.5% (T1), 0.75% (T2), 1.0% (T3), 1.25% (T4), and 1.5% (T5). Statistically significant differences between treatments are indicated by different lowercase letters based on the Tukey test (*p* < 0.05; *n* = 6).

Treatments	CK	T1	T2	T3	T4	T5
Total root length (cm)	274.69 ± 6.43 c	349.09 ± 12.88 b	412.95 ± 17.15 a	335.16 ± 12.05 b	361.38 ± 12.66 ab	327.59 ± 11.42 bc
Total root surface area(cm^2^)	23.51 ± 2.56 e	36.71 ± 1.52 cd	53.99 ± 1.96 b	62.21 ± 1.23 a	29.42 ± 1.82 de	37.21 ± 0.96 c
Total root projection area (cm^2^)	9.67 ± 1.76 b	18.07 ± 0.95 a	20.14 ± 0.6 a	12.39 ± 0.56 b	9.06 ± 0.56 b	11.24 ± 0.62 b
Average root diameter (mm)	0.39 ± 0.02 b	0.37 ± 0.02 b	0.43 ± 0.01 ab	0.47 ± 0.01 a	0.41 ± 0.02 ab	0.40 ± 0.01 b
Root volume (cm^3^)	0.29 ± 0.04 b	0.35 ± 0.04 b	0.63 ± 0.01 a	0.51 ± 0.03 a	0.31 ± 0.02 b	0.36 ± 0.01 b

## Data Availability

The datasets generated for this study are available on request to the corresponding author.

## Abbreviations

The following abbreviations are used in this manuscript:

EC	Electrical conductivity
TN	Total nitrogen
TP	Total phosphorus
TC	Total carbon
TOC	Total organic carbon
